# The Care and Feeding of Children

**Published:** 1898-03

**Authors:** 


The Care and Feeding of Children. A catechism for the use of mothers and children's nurses. By L. Emmett Holt, M. D., Professor of Diseases of Children in the New York Polyclinic; Attending Physician to the Babies' Hospital and the Nursery and Child's Hospital, New York. Second edition, revised and enlarged. Duodecimo, pp. 104. New York: D. Appleton & Co. 1897.

This little volume in its first edition was a work of much value in the training of children's nurses. The thorough revision and enlargement which the author has given the present edition has much increased its usefulness. The physician will find it an excellent work to put into the hands of any mother, for it tells just enough and tells it so clearly that no one can fail to understand. The space devoted to infant feeding is much greater than in the first edition and the teaching is thoroughly up-to-date.

